# Redução de Sal na Dieta: Ilusão ou Realidade?

**DOI:** 10.36660/abc.20200155

**Published:** 2020-04-06

**Authors:** Nereida Kilza da Costa Lima

**Affiliations:** 1 Universidade de São Paulo Campus de Ribeirão PretoRibeirão PretoSPBrasilUniversidade de São Paulo Campus de Ribeirão Preto, Ribeirão Preto, SP – Brasil

**Keywords:** Hipertensão/prevenção e controle, Países em Desenvolvimento, Adesão a Medicação, Cloreto de Sódio na Dieta, Doenças Cardiovasculares/mortalidade

Apesar do amplo conhecimento da hipertensão arterial sistêmica como principal fator de risco para doenças cardiovasculares, as taxas de controle apresentam modesto aumento progressivo, especialmente no Brasil. Estudos brasileiros evidenciam que 50% a 60% dos hipertensos estão sob tratamento e apenas 20% a 30% do total de hipertensos estão controlados.^1^

A maior dificuldade em controlar a hipertensão arterial sistêmica está em conseguir a adesão dos pacientes a longo prazo, pois a maioria é assintomática e, eventualmente, inicia sintomas decorrentes do uso de medicações anti-hipertensivas.

Se a adesão aos medicamentos é baixa, menor ainda é a adesão às mudanças no estilo de vida. Entre estas mudanças, reduzir o sal na dieta tem se apresentado como um grande desafio. É indiscutível o benefício da redução moderada do sal na redução da pressão arterial, especialmente para hipertensos, além dos efeitos na prevenção de eventos cardiovasculares, ainda que esta redução seja de pelo menos um terço do sal ingerido habitualmente ou almejando o total de 5 gramas de sal por dia, segundo a OMS.^2^ O consumo de sal atual é muito elevado, principalmente entre os hipertensos, variando de 9 a 12 gramas por dia.^3^

Entre as diferentes estratégias para que se consiga a redução de sal na dieta, a mais comumente empregada pela equipe multidisciplinar, incluindo o médico, consiste na orientação para evitar alimentos industrializados (embutidos, enlatados, etc.), preferindo os alimentos não processados, associada à redução do sal no preparo das refeições e à retirada do saleiro da mesa.^4^ Também é frequente a orientação do uso de temperos como alho, cebola e orégano, que podem melhorar o paladar dos alimentos, havendo menor necessidade de utilização do sal. Em estudo recente, verificou-se que o uso de orégano associado à massa do pão comum mudou a preferência de hipertensos e normotensos, jovens ou idosos, para consumo de pães com menor teor de sódio, por melhorar o paladar.^5^ No entanto, manter esta preferência por longo tempo é um grande desafio e esta intervenção ainda não foi testada. Indivíduos de meia-idade que utilizaram de forma randomizada e cruzada pães com sal reduzido (0,3 g de sal por 100 g) ou pães com sal habitual (1,2 g de sal por 100 g) apresentaram queda da pressão arterial sistólica e queda da excreção urinária de sódio após 5 semanas de consumo de pães com sal reduzido, quando comparados ao momento após 5 semanas de pão habitual.^6^

O que se observa, em geral, quando são orientadas as mudanças de estilo de vida, é que a adesão inicial é boa, mas as mudanças não permanecem ao longo do tempo. Acreditar que o paciente é o único responsável pela má adesão não é correto. A vida moderna trouxe o hábito das refeições fora de casa, com pouco tempo disponível, além do sal, atualmente, ser o conservante mais utilizado pela indústria alimentícia. Mesmo com orientação individualizada, dentro de um protocolo de pesquisa bem estruturado, onde todo sal de adesão era entregue embalado, Arantes et al.,^7^não observaram redução da quantidade total de sal ingerida por voluntários de meia-idade, todos funcionários de uma universidade pública, ao longo de três meses de seguimento. Apesar da orientação de que no mínimo quatro refeições principais fossem feitas em domicílio, além da importância de escolher alimentos com menos sal, provavelmente a quantidade total de sódio excretada em 24 horas, que estima a quantidade de sal ingerido no mesmo período, não foi reduzida por ter ocorrido consumo de alimentos mais salgados em refeições realizadas fora da casa dos voluntários ou mesmo por escolha de alimentos mais salgados no próprio domicílio. Foi possível verificar a associação entre maior excreção de sal nos hipertensos com maior pressão arterial diastólica central e na medida casual.

As ações até agora implementadas para a redução da ingestão de sal em alimentos industrializados propiciaram redução de 17 toneladas de sal nos alimentos entre 2011 e 2016, principalmente nas misturas para sopas ou nas sopas instantâneas, linguiças, queijos e requeijões. Em 2017, um novo acordo entre o Ministério da Saúde e as indústrias alimentícias teve como alvo a redução de sal em pães e massas instantâneas.

Reduzir o sal dos alimentos processados, sem comprometer o sabor e sem prejudicar a conservação deles, torna o trabalho da indústria complexo. Mas a redução de sal nos alimentos industrializados precisa avançar, assim como a educação da população. Há que se considerar que os indivíduos já habituados ao maior consumo de sal possam adicionar sal aos alimentos industrializados se considerarem que eles fiquem mais saborosos desta forma e não estiverem cientes dos riscos associados a esta prática.

Um interessante estudo holandês realizou uma simulação de duas estratégias diferentes para a redução do consumo de sal, com alvo em até 6 gramas de sal por dia, baseada em dados da população nacional. Uma das estratégias seria a substituição de alimentos com mais sal por outros similares, mas com menos sal, já existentes no mercado, enquanto a outra pressupunha a mudança do conteúdo de sal de alimentos processados dentro do que seria possível. Foi observado que a redução do consumo de sal, com qualquer uma destas estratégias, seria de cerca de 30%, com queda da pressão arterial sistólica de 1,6 mmHg e potencial redução de incidência de infarto agudo do miocárdio de 4,8%.^8^

É importante a educação para a valorização das medidas saudáveis. Ainda temos, infelizmente, uma dissociação entre o que é bom para a saúde e o que é melhor aceito pela sociedade, principalmente quando observamos o comportamento de jovens em festas ou finais de semana, quando ainda se deparam com a dificuldade em ingerir menor quantidade de bebidas alcoólicas e preferir alimentos mais saudáveis, sem sofrer discriminação.

Um estudo italiano identificou que tanto o conhecimento sobre a ingestão de sal (alimentos com mais sal, habilidade de ler rótulos, etc.) como a prática deste conhecimento eram reduzidos principalmente em adolescentes e indivíduos com escolaridade mais baixa.^9^

A educação para o menor consumo de sal terá que receber amplo apoio de instâncias governamentais, indústrias, escolas, profissionais da saúde e publicitários, para que seja criada uma cultura diferente da atual. Este processo tem que começar na infância, mas toda a família precisa ser integrada e os idosos de uma comunidade podem ser importantes veiculadores de mudanças de hábitos.

Avaliando as estratégias para redução do sal em países de todas as regiões do mundo, um estudo identificou que as regiões com menores iniciativas eram a África, o sudeste asiático e o Mediterrâneo oriental.10 Somente a implementação de várias estratégias para a redução do consumo de sal, de forma concomitante e organizada, com monitoramento dos efeitos, poderá ter impacto real na redução das doenças cardiovasculares.

Portanto, a redução de sal na dieta é possível se realmente for um objetivo das políticas nacionais e regionais de saúde e educação. Entretanto, quando a realidade ainda parece distante, apesar dos esforços serem crescentes, o que permanece é a sensação de algo ilusório.
