# Supplementary material for: Redução de Sal na Dieta: Ilusão ou Realidade?
Source: Arq Bras Cardiol. 2020 Apr 6;114(3):562–3. [Article in Portuguese] doi: 10.36660/abc.20200155 (PMC7792740; doi:10.36660/abc.20200155)
Supplement: Translation

# Translation: Dietary Salt Reduction: Illusion or Reality?

**Authors:** Nereida Kilza da Costa Lima

**Affiliations:** 1 BrazilUniversidade de São Paulo Campus de Ribeirão Preto, Ribeirão Preto, SP – Brazil

**Keywords:** Hypertension/prevention and control, Developing Countries, Medication Adherence, Sodium Chloride, Dietary, Cardiovascular Diseases/mortality

In spite of ample knowledge regarding systemic arterial hypertension as the main risk factor for cardiovascular diseases, the rates of control have shown a modest progressive increase, especially in Brazil. Brazilian studies have provided evidence that 50% to 60% of people with hypertension are receiving treatment, while only 20% to 30% of all people with hypertension are controlled.^1^

The main difficulty in controlling systemic arterial hypertension lies in managing long-term patient adherence, given that the majority of patients are asymptomatic, and they eventually begin to have symptoms as a result of antihypertensive medication use.

While adherence to medical treatment is low, adherence to lifestyle changes is even lower. Among these changes, dietary salt reduction has posed a major challenge. The benefits of moderate salt reduction to blood pressure, especially for patients with hypertension, are undeniable, as are its effects on preventing cardiovascular events, even if the reduction is by at least one third of the salt regularly ingested or a goal of no more than 5 grams of salt daily, according to the World Health Organization.^2^ Current salt consumption is very high, especially among patients with hypertension, ranging from 9 to 12 grams daily.^3^

Among different strategies for successfully reducing dietary salt, the one most commonly employed by multidisciplinary teams, which include a physician, consists of advising patients to avoid processed foods (sausages, canned foods, etc.), giving preference to unprocessed foods, in combination with reducing salt when preparing meals and removing the salt shaker from the table.^4^ The use of spices such as garlic, onion, and oregano is also frequently recommended, given that they may enhance the taste of food, thus lowering the need to use salt. A recent study found that the use of oregano in common bread dough changed the preferences of young and elderly hypertensive and normotensive individuals with respect to consumption of reduced-sodium bread, by improving the flavor.^5^ Maintaining this preference over a long time, however, is a major challenge, and this intervention has not yet been tested. Middle-aged individuals who, in a randomized cross-over manner, used low-salt bread (0.3 g of salt per 100 g) or bread with the conventional level of salt (1.2 g of salt per 100 g) showed a decrease in systolic blood pressure and a decrease in urine excretion of sodium after 5 weeks of consuming the low-salt bread, when compared to the moment 5 weeks after consuming the conventional bread.^6^

When patients are advised to make lifestyle changes, what is generally observed is that initial adherence is good, but the changes do not continue over time. It is not correct to assume that patients are the only ones responsible for poor adherence. Modern life has led to habits of eating meals outside of the home, with little time available; salt is, furthermore, the preservative most widely used by the food industry. Even with individualized advice within a well structured protocol, where all the salt for adherence was given in a package, Arantes et al.^7^ did not observe a reduction in the total quantity of salt ingested by middle-aged volunteers, all of whom were employs of a public university, over three months of follow-up. Notwithstanding advice to prepare at least four main meals at home, in addition to advice regarding the importance of choosing foods with less salt, the total quantity of sodium excreted in 24 hours, which estimates the quantity of salt ingested during the same period, was probably not reduced due to the consumption of saltier foods outside of the house or even due to the choice of saltier foods at home. It was possible to verify an association of greater salt excretion in hypertensive patients with higher central diastolic blood pressure and casual measurement.

The actions implemented to reduce salt intake in processed foods so far have promoted the reduction of 17 tons of salt in foods between 2011 and 2016, especially in mixtures for soup, instant soup, sausage, cheese, and cottage cheese. In 2017, the target of a new agreement between the Ministry of Health and the food industries was to reduce salt in bread and instant pasta.

Reducing the quantity of salt in processed foods, without compromising their taste or jeopardizing their preservation, makes the industry’s work complex, but the reduction of salt in processed foods needs to advance, as does the education of the population. It is necessary to consider that individuals who are already used to higher salt consumption may add salt to processed foods if they consider that doing so makes these foods taste better and if they are unaware of the risks associated with this practice.

An interesting Dutch study performed a simulation of two different strategies for reducing salt consumption, with a goal of up to 6 grams of salt daily, based on data from the Dutch population. One of the strategies would be substituting high-salt foods with similar low-salt foods that are already commercially available, while the other proposed reducing the salt content of processed foods to the extent that it was possible. They observed that the reduction in salt consumption, with either of these strategies, would be approximately 30%, decreasing systolic blood pressure by 1.6 mmHg, with a potential 4.8% reduction in the incidence of acute myocardial infarction.^8^

Education that promotes healthy measures is important. Unfortunately, there is still a lack of association between what is good for our health and what is most accepted by society, especially when we observe young people’s behavior at parties or on weekends, when they further face the difficulty of ingesting lower amounts of alcoholic beverages or giving preference to healthier foods without suffering discrimination.

An Italian study found that both knowledge regarding salt ingestion (foods with more salt, the habit of reading labels, etc.) and behavior based on this knowledge were primarily lower in adolescents and individuals with lower levels of schooling.^9^

Education for lower salt consumption will have to be widely supported by government agencies, industries, schools, healthcare professionals, and the advertising industry, in order to create a culture different from the current one. This process will have to start in early childhood, but the whole family will need to be integrated, and elderly people may be important agents of habit change within their communities.

Evaluating strategies for salt reduction in different countries in all world regions, one study identified that the regions with the fewest initiatives were Africa, South East Asia, and the Eastern Mediterranean.^10^ Only the implementation of diverse strategies for reducing salt conception, in a concomitant and organized manner, as well as the monitoring of their effects, will be able to have a real impact on the reduction of cardiovascular diseases.

Dietary salt reduction will therefore be possible when it truly becomes the objective of national and regional health and education policies. As long as this reality, however, appears to be far off, even though efforts are growing, what remains is the impression of an illusion.
